# Comparing effects of low and high-flow anesthesia on hemorheology and coagulation factors

**DOI:** 10.12669/pjms.313.6320

**Published:** 2015

**Authors:** Orhan Binici, Ismail Kati, Ugur Goktas, Lokman Soyaral, Osman Cagatay Aytekin

**Affiliations:** 1 Orhan Binici, MD. Anesthesiology and Reanimation, Erzincan State Hospital, Erzincan, Turkey; 2 Ismail Kati, MD. Yuzuncuyil University Medical Faculty, Anesthesiology and Reanimation, Van, Turkey; 3 Ugur Goktas, MD. Yuzuncuyil University Medical Faculty, Anesthesiology and Reanimation, Van, Turkey; 4 Lokman Soyaral, MD. Yuzuncuyil University Medical Faculty, Anesthesiology and Reanimation, Van, Turkey; 5 Osman Cagatay Aytekin, MD. Yuzuncuyil University Medical Faculty, Anesthesiology and Reanimation, Van, Turkey

**Keywords:** Hemorheology, Cogulation, Low flow anesthesia

## Abstract

**Objectives:**

In the current study, we compared the effects of low- and high-flow anesthesia techniques on hemorheology and coagulation parameters in patients who received sevofluran.

**Methods:**

Forty patients classified as Risk Group I–II according to American Society of Anesthesiologists’ (ASA) guidelines who were scheduled to undergo general anesthesia were randomly assigned to one of two groups. Low-flow anesthesia was administered to the first group, and high-flow anesthesia was used in the second group. Blood samples were obtained in the preoperative and peroperative periods (at 60 and 120 min) for determination of blood and plasma viscosity, plasma oncotic pressure, international normalized ratio (INR), phorotrombin time (PT), activated partial phorotrombin time (aPTT) and fibrinogen. Blood was also drawn for analysis of factor VIII (FVIII) activity, which was measured in the preoperative period and at postoperative six hour.

**Results:**

The peroperative plasma viscosity was significantly low in Group 1 relative to Group 2. aPTT was significantly elevated at 60 minutes in Group 1 relative to Group 2, but the increase at 120 minutes was not significant.

**Conclusion:**

The effects of low-flow anesthesia on hemorheology were greater than those of high-flow anesthesia.

## INTRODUCTION

Hemorheology deals with functions and inter-relations of in vivo blood, blood cells, and blood vessels. Hemorheological disorders may lead to hypoxia at the tissue level. Dysfunction has adverse influences on the microcirculation of those tissues.^1^ Plasma proteins, such as albumin, globulin, and fibrinogen, affect the viscosity of plasma. Several studies have demonstrated that anesthetic agents carried by blood to a target organ affect the viscosity of the blood by changing that of plasma during transportation and influencing the smooth muscle of the vessels, resulting in vasodilatation.^2^-^3^ Studies have reported that changes in hemorheological factors are involved in the pathophysiology or prognosis of many diseases.^4^ Sevofluran is commonly used for general anesthesia. As this agent influences hemorheological parameters, it is very important in patients with microcirculation disorders, bleeding diathesis, and coagulopathy. To avoid exposure to liquid parameters in previous studies, patients who underwent the same surgical procedures received standard therapy and standard induction agents.^5^,^6^ Although some articles in the literature have examined the effects of sevofluran on hemorheology during general anesthesia, none have investigated its effect on hemorheology and coagulation factors. We believe that the current study will be instructive in this issue.

## METHODS

The approval of the local ethics committee was obtained, in addition to written and verbal consent from the patients. Forty patients (age range: 20–64 years) were enrolled who were classified as Risk Group I–II according to American Society of Anesthesiologists’ (ASA) guidelines. This was a randomized, prospective, and single-blinded study. Conditions requiring a blood transfusion, patients with abnormal preoperative laboratory parameters (electrolyte imbalance, anemia, polycytemia, thrombocytopenia, leucopenia, leukocytosis), patients with a known disorder (e.g., diabetes, hematological diseases), patients taking anticoagulant and/or antiaggregating agents, patients with long-term starvation, dehydrated or extremely hydrated patients, and ASA Group III–IV patients were excluded.

Electrocardiogram traces were obtained, and the systolic blood pressure (SBP), diastolic blood pressure (DBP), mean blood pressure (MBP), peripheral oxygen saturation (SpO2), and end-tidal CO2 were monitored (Datex-Ohmeda, Finland) before the patients underwent surgery.

Intravenous access was made with a 20G IV cannula inserted into the dorsum of the hand, and 0.9% sodium chloride was infused at 2 mL/kg/h. The infusion rate was not changed unless it was necessary. Premedication with 0.03 mg/kg of midazolam was administered in the operating room. The right radial artery was then cannulated under local anesthesia, and baseline blood samples were drawn. Anesthesia was induced with 2 mg/kg of propofol, 0.1 mg/kg of vecuronium, and 2 µg/kg of fentanyl. The patients were intubated following the induction of anesthesia. They were randomized into two groups based on the patient’s order in the operation list. Low-flow anesthesia (1 L/min) was administered to the first group, and high-flow anesthesia (6 L/min) was used in the second group. Anesthesia with sevofluran was maintained with an end-tidal concentration of 1.5–2%, a mixture of N2O and oxygen (1:1), 2 mg of vecuronium (once in 30 minutes), and 50 µg of fentanyl. Mechanical ventilation in the volume-controlled mode was performed with end-tidal CO2 of 30–35 mmHg.

The blood samples were obtained at different points, including immediately before the operation and 60 and 120 minutes after the procedure for the determination of blood and plasma viscosity, plasma oncotic pressure, INR, PT, aPTT and fibrinogen. An additional two samples were drawn immediately before the operation and at postoperative 6 hour for determination of factor VIII. SBP, DBP, MBP, SpO2, and body temperature were recorded once every 5 minutes. The blood samples were drawn from the radial artery cannula for the analysis of plasma viscosity, plasma oncotic pressure, fibrinogen, international normalized ratio (INR), phorotrombin time PT, activated partial phorotrombin time (aPTT) and factor VIII (FVIII).

The viscosity of the blood and plasma samples was measured with a Wells-Brookfield cone-plate rotational viscometer (Brookfield DV-II, USA) at 37°C. The viscosity of the blood was measured at different shear rates in vessels with three different diameters (large K1, middle K2, and capillary K3) to examine blood rheology. For the measurement of plasma viscosity, the blood samples were centrifuged at 3000 rpm for 15 minutes to separate the blood cells. All the blood viscosity results were statistically standardized for K1 (243), K2 (110), and K3 (23) hematocrit factors. The plasma viscosity was measured at three different shear rates (shear rates: 23, 110, and 243). The arithmetical means of the results were calculated, and the plasma viscosity was determined. The results are expressed in centi-poise (cP).

The plasma oncotic pressure was measured as mmHg with an Osmomat 050 Colloid Osmometer Viscometer (Gonotec GmbH, Germany). Fibrinogen, PT, aPTT, and FVIII were analyzed in the hematology laboratory with a fully automated coagulation device (STA, France), and Hct was measured in the hematology laboratory.

Definitive statistics for continuous variables were expressed as the mean±standard deviation, and categorical variables were expressed as figures. The Student’s *t* test was used to compare the groups with regard to age, gender, and ASA in terms of proportions in the groups compared with the *Z* test (comparison rate).

A two-factor analysis of variance (ANOVA) and two-factor repeated measures ANOVA of one factor were conducted to determine whether there was a difference between the drugs and measurement points for the continuous variables. The blood viscosity value was considered a covariable for Hct, and statistical correction was done with repeated measures covariance.

Following the ANOVA, Tukey’s multiple comparison test differences in the studied parameters at different times were assessed with a two-factor analysis of variance (ANOVA) and two-factor repeated measures ANOVA of one factor. Pearson’s correlation coefficient was measured separately in the two groups to determine the relation between the studied parameters. *P*<0.05 was considered statistically significant in the calculations.

## RESULTS

There were no significant between-group differences in demographics (Table-I). The plasma viscosity values are given in Table-II. Plasma viscosity values were reduced in Group 1 relative to Group 2 at 60 and 120 minutes (*P*<0.05). The K1, K2, and K3 blood viscosity values are given in Table-II. No difference was found in the intergroup comparison (*P*>0.05). The oncotic pressure values and FVIII values are presented in Table-III. As shown in the table, the preoperative oncotic pressure was higher in Group 2 than Group 1 (*P*<0.05). Intra- and intergroup comparisons revealed no significant differences in the FVIII values of the groups (*P*>0.05). The fibrinogen, aPTT, PT, and thrombocyte count values of the groups are given in Table-IV. There was no change in the fibrinogen values in the intergroup comparison (*P*>0.05). However, aPTT values were higher in Group 1 at 60 minutes relative to Group 2 (*P*<0.05). There were also no significant differences in the intergroup comparisons of PT (*P*>0.05). The preoperative and 120 minutes thrombocyte values were significantly reduced in both groups (*P*<0.05).

**Table-I:** Demographics of the groups (Mean ± SD).

	Group 1 (*n*=20)	Group 2 (*n*=20)
Age (year)	33.13±12.55	35.13±12.17
Gender (male/female)	13/7	10/10
ASA	13/7	12/8

**Table-II:** Plasma and blood viscosity values of the groups (Mean ± SD).

Viscosity (cP)		Group 1 (n=20)	Group 2 (n=20)
K1 blood viscosity	Preop.	4.60±0.98	4.57±0.56
	60 min	4.21±0.91	3.84±0.36 #
	120 min	3.84±0.65 Δ	3.75±0.40 #
K2 blood viscosity	Preop.	4.47±0.82	4.91±0.74
	60 min	3.99±0.70	4.09±0.53 #
	120 min	3.87±0.71 Δ	3.99±0.58 #
K3 blood viscosity	Preop.	5.25±1.87	6.00±1.43
	60 min	4.55±1.68 Δ	4.85±1.31 #
	120 min	4.52±1.60 Δ	4.74±1.34 #
Plasma viscosity	Preop.	1.51±0.35	1.51±0.24
	60 min	1.31±0.21	1.46±0.28 *
	120 min	1.23±0.26 Δ	1.40±0.31 # *

In the table,

* indicates the intergroup comparison,

Δ indicates the intragroup comparison of Group 1, and

# indicates the intragroup comparison of Group 2 (P<0.05). High shear rate (K1); moderate shear rate (K2); low shear rate (K3); cP: centipoise; preop.: preoperative.

**Table-III:** Oncotic pressure and FVIII values of the groups (mean±SD).

	Group 1 (n=20)	Group 2 (n=20)
Preoperative oncotic pressure (mmHg)	20.01±1.71	21.85±1.83 *
Oncotic pressure at 60 min (mmHg)	18.14±2.06Δ	17.59±1.92 #
Oncotic pressure at 120 min (mmHg)	17.55±1.82 Δ	16.95±1.58 #
Preoperative FVIII	0.54±0.17	0.56±0.46
FVIII at postoperative 6 h	0.59±0.23	0.51±0.26

In the table,

* indicates the intergroup comparison (P<0.05),

Δ indicates the intragroup comparison of Group 1, and

# indicates the intragroup comparison of Group 2 (P<0.05).

**Table-IV:** aPTT, PT, INR, and fibrinogen values of the groups (Mean ± SD).

	Group I (n=20)	Group 2 (n=20)
Preoperative aPTT	29.82±2.17	29.67±2.29
aPTT at 60 min	31.35±3.56 *	28.63±3.94
aPTT at 120 min	31.80±3.92	31.80±3.92
Preoperative PT	13.03±1.09	13.29±1.15
PT at 60 min	13.91±1.04 Δ	13.76±0.86 #
PT at 120 min	13.69±1.12 Δ	13.88±1.15 #
Preoperative thrombocyte count	295.95±69.08	293.50±97.03
Thrombocyte count at 60 min	279.55±90.03	279.10±105
Thrombocyte count at 120 min	263.05±70.51 Δ	263.10±81.52 #
Preoperative fibrinogen (mg/dL)	321.10±112	312.40±154.83
Fibrinogen (mg/dL) at 60 min	290.80±90.92 Δ	299.85±169.56 #
Fibrinogen (mg/dL) at 120min	296.10±79.87 Δ	296.05±157.35 #

In the table,

* indicates the intergroup comparison (P<0.05),

Δ indicates the intragroup comparison of Group 1, and

# indicates the intragroup comparison of Group 2 (P<0.05).

## DISCUSSION

Hemorheology is influenced by the type of anesthesia (general or regional), blood pressure, type of anesthetic agent, duration of the operation, volume of bleeding, duration of bleeding, infusion of crystalloid or colloidal fluids, body weight, hypothermia, hyperthermia, Hct, Hb, albumin, cholesterol, fibrinogen, and total protein.^7^,^8^

Anesthetic drugs may alter the diameters of arterioles and venules, as well as the response of those structures to stress. During general anesthesia, the blood flow of the popliteal and external iliac veins is reduced by 50% secondary to sympathetic inactivation, increasing the likelihood of thrombosis occurring.^9^ A previous study examined outcomes in general anesthesia (high-flow anesthesia) administered for patient-controlled IV analgesia (morphine) and combined spinal epidural anesthesia administered for patient-controlled epidural analgesia to patients who were scheduled to undergo knee arthroplasty. The study reported that complete blood viscosity levels were significantly elevated in the general anesthesia group at postoperative 24 and 48 h.^3^ In the current study, low-flow anesthesia significantly reduced peroperative plasma viscosity in comparison with high-flow anesthesia.

A study of the influence of high-flow anesthesia with propofol, fentanyl, and vecuronium on the aggregation of platelet (PLT) in subjects given sevofluran or sevofluran and propofol showed that intraoperative and early postoperative PLT aggregation decreased relative to preoperative values in those given sevofluran alone.^10^ In another study, sevofluran decreased PLT functions by suppressing thromboxane A2 in high-flow anesthesia.^11^ In spinal and general anesthesia of pregnant women, PT, aPTT, and fibrinogen parameters showed no significant intergroup changes, but the PLT count and hematocrit level were significantly reduced in comparison to the preoperative values of both groups.^12^ In the current study, there were no differences between-group in PLT values, but there was a statistically nonsignificant reduction in these values in the peroperative period relative to the preoperative period in both groups. There were no between-group differences in aPTT and PT values in the current study, although peroperative PTT and PT values showed a statistically nonsignificant increase relative to preoperative values in the intergroup comparisons. This finding is possibly related to low intraoperative blood loss and peroperative hydration.

In a study that investigated the effects of body temperature, shear rate, and different volume expanders on blood viscosity, when body temperature was reduced from 37°C to 22°C, the blood viscosity increased from 50% up to 300%. Viscosity values were high at body temperatures below 15°C and a low shear rate, with a higher shear rate associated with lower viscosity.^13^ In the current study, plasma and blood viscosity values were measured at different shear rates in both groups while keeping the temperature constant. The resultant values are compatible with the literature with plasma and blood viscosity being low at high shear rates.

In a study of plasma oncotic pressure and total proteins, colloidal oncotic pressure and total protein values were significantly low in comparison with preoperative values but there was no change in a control group.^14^ In the current study, peroperative oncotic pressure values were significantly reduced in both groups relative to preoperative oncotic pressure values. This finding is possibly related to peroperative hydration with saline in both groups.

Studies have reported that sympathetic stimulation significantly increases factor VIII and von Willebrand factor, reduces levels of antithrombin III, and induces platelet aggregation.^15^,^16^ General or epidural anesthesia was administered to a premenopausal patient who was scheduled to undergo hysterectomy, and changes in coagulation and fibrinolysis were investigated. The researchers reported an increase in FVIII and the fibrinopeptide A complex with both anesthesia methods.^17^ In the current study, intra and intergroup comparisons revealed no significant differences in the FVIII values of the groups. There were no between-group differences in fibrinogen values, but there was a statistically nonsignificant reduction in these values in the peroperative period relative to the preoperative period in both groups. The latter was attributed to peroperative hydration.

In conclusion, the current study showed that low-flow anesthesia influenced hemorheology more than high-flow anesthesia since there was no difference between groups in terms of oncotic pressure, blood viscosity, bleeding and fluids infused, and plasma viscosity was low and aPTT was high. We believe that further studies are required to clarify this issue.
